# Mechanism-aware imputation: a two-step approach in handling missing values in metabolomics

**DOI:** 10.1186/s12859-022-04659-1

**Published:** 2022-05-16

**Authors:** Jonathan P. Dekermanjian, Elin Shaddox, Debmalya Nandy, Debashis Ghosh, Katerina Kechris

**Affiliations:** Department of Biostatistics and Informatics, Colorado School of Public Health, University of Colorado Anschutz Medical Campus, Aurora, CO USA

**Keywords:** Missing data, Imputation, Machine learning, Metabolomics

## Abstract

When analyzing large datasets from high-throughput technologies, researchers often encounter missing quantitative measurements, which are particularly frequent in metabolomics datasets. Metabolomics, the comprehensive profiling of metabolite abundances, are typically measured using mass spectrometry technologies that often introduce missingness via multiple mechanisms: (1) the metabolite signal may be smaller than the instrument limit of detection; (2) the conditions under which the data are collected and processed may lead to missing values; (3) missing values can be introduced randomly. Missingness resulting from mechanism (1) would be classified as Missing Not At Random (MNAR), that from mechanism (2) would be Missing At Random (MAR), and that from mechanism (3) would be classified as Missing Completely At Random (MCAR). Two common approaches for handling missing data are the following: (1) omit missing data from the analysis; (2) impute the missing values. Both approaches may introduce bias and reduce statistical power in downstream analyses such as testing metabolite associations with clinical variables. Further, standard imputation methods in metabolomics often ignore the mechanisms causing missingness and inaccurately estimate missing values within a data set. We propose a mechanism-aware imputation algorithm that leverages a two-step approach in imputing missing values. First, we use a random forest classifier to classify the missing mechanism for each missing value in the data set. Second, we impute each missing value using imputation algorithms that are specific to the predicted missingness mechanism (i.e., MAR/MCAR or MNAR). Using complete data, we conducted simulations, where we imposed different missingness patterns within the data and tested the performance of combinations of imputation algorithms. Our proposed algorithm provided imputations closer to the original data than those using only one imputation algorithm for all the missing values. Consequently, our two-step approach was able to reduce bias for improved downstream analyses.

**Supplementary Information:**

The online version contains supplementary material available at 10.1186/s12859-022-04659-1.

## Background

Metabolomics refers to the comprehensive profiling of metabolite abundances, which are typically measured using mass spectrometry (MS) or nuclear magnetic resonance (NMR) spectrometry [1]. While the two most common mass spectrometry approaches in metabolomics are gas chromatography coupled with mass spectrometry (GC–MS) and liquid chromatography coupled with mass spectrometry (LC–MS) [2], these metabolomics instruments often introduce missing values into the data. Missingness occurs via four mechanisms: (1) the metabolite signal may be smaller than the detection limit of the instrument, (2) the environment under which measurements are generated, such as the batch, the specific instrument used, or the variation in bioinformatics processing pipelines, may lead to missing values, (3) missing values can be introduced randomly, and (4) the metabolite is not present outright in a sample for biological or environmental reasons.

These mechanisms for missing data can be described in the context of three general frameworks. Sparsity resulting from mechanism (1), (2), and (3) are referred to as Missing Not At Random (MNAR), Missing At Random (MAR), and Missing Completely At Random (MCAR) [3], respectively. MCAR values are independent of both the observed and missing values and arise randomly. MAR values are independent of missing values but are dependent on the observed values (e.g., measured hormone variable). This type of missingness can also arise in metabolomics due to suboptimal data preprocessing [4]. If the missing values are dependent on a factor/covariate that we do not observe, then it is MNAR. This missingness type most often arises from metabolite signals being below the limit of detection of a particular instrument. In practice, metabolomics data are known to contain a mixture of MAR, MCAR and MNAR missing data [4] which are typically omitted from the data set for further analyses, or otherwise, they are imputed. However, omitting missing data that are not MCAR may reduce statistical power for downstream analyses [3]. On the other hand, if missing values are imputed poorly, we risk introducing bias into our results [3].

A technique for omitting missing data is to assess whether multivariate data missing values are MCAR or not, before omitting the values [5]. However, the limitation of this approach is that if missing values are indeed MCAR, then dropping observations would still result in reduced statistical power, and if the missing values are not MCAR then one must resort to some alternative solution. Moreover, this procedure naïvely attempts to discretize multiple missing values into two categories where all the missing data are either MCAR or not MCAR. An alternative to handling missing data in the analysis is to handle it via study design [6]. For example, one might design a study that attempts to avoid/minimize the number of missing values generated by excluding individuals that may have a higher probability of dropping out. However, this approach may be infeasible, and such a strategy might lead to biased findings for the population of interest, due to selection bias. A potential solution to avoid dropping observations is imputation of missing data. However, imputation algorithms are typically specific to missing mechanisms [4]. For instance, an imputation algorithm may assume all missingness is MNAR when estimating values [4, 7–9]. Therefore, applying an incorrect imputation algorithm may produce data that are not representative of the true unobserved data.

In recent years, many imputation algorithms have been developed to estimate data that are missing, but they tend to be optimal for a particular missing mechanism [4]. Commonly, MAR/MCAR values are grouped together since they are difficult to distinguish in practice. MAR/MCAR and MNAR values are best imputed with different algorithms [4]. Typically, algorithms that are used to impute MAR/MCAR values include neighbor-based algorithms such as K-nearest neighbors (KNN), probabilistic estimating algorithms such as Bayesian principal component analysis (BPCA), and regression-based methods such as random forest imputation [4, 10]. On the other hand, algorithms that are used for MNAR value imputation include the neighbor-based no-skip KNN (nsKNN), where neighbors (of a target sample) with shared missing values for the same metabolite are assumed to be MNAR and estimated as the minimum of that metabolite being imputed. MNAR values can also be imputed using regression-based quantile regression imputation of left-censored data (QRILC), or regression-based linear regression models for randomly censored covariates [7–9]. Ni et al. [4] discovered that random forest imputation resulted in the most accurate estimation of MAR/MCAR values, whereas QRILC resulted in the most accurate estimation of MNAR values. However, since these imputation algorithms perform best on specific types of missing data mechanisms, the effectiveness of imputation will depend on the ratio of MAR/MCAR to MNAR, which is unknown in practice.

In this work we propose a novel Mechanism-Aware Imputation (MAI) algorithm that relies on first predicting the missing mechanism (MAR/MCAR versus MNAR) using a Random Forest classifier [11]. Once missing values are classified, we apply the most appropriate existing imputation algorithm specific to that predicted missing mechanism. Our simulations demonstrate that MAI results in a better approximation to the true (unobserved) data, and results in less biased imputed values. Consequently, this can help downstream data analyses yield higher statistical powers, and more reliable conclusions than other imputation approaches.

## Methods

**Fig. 1:**
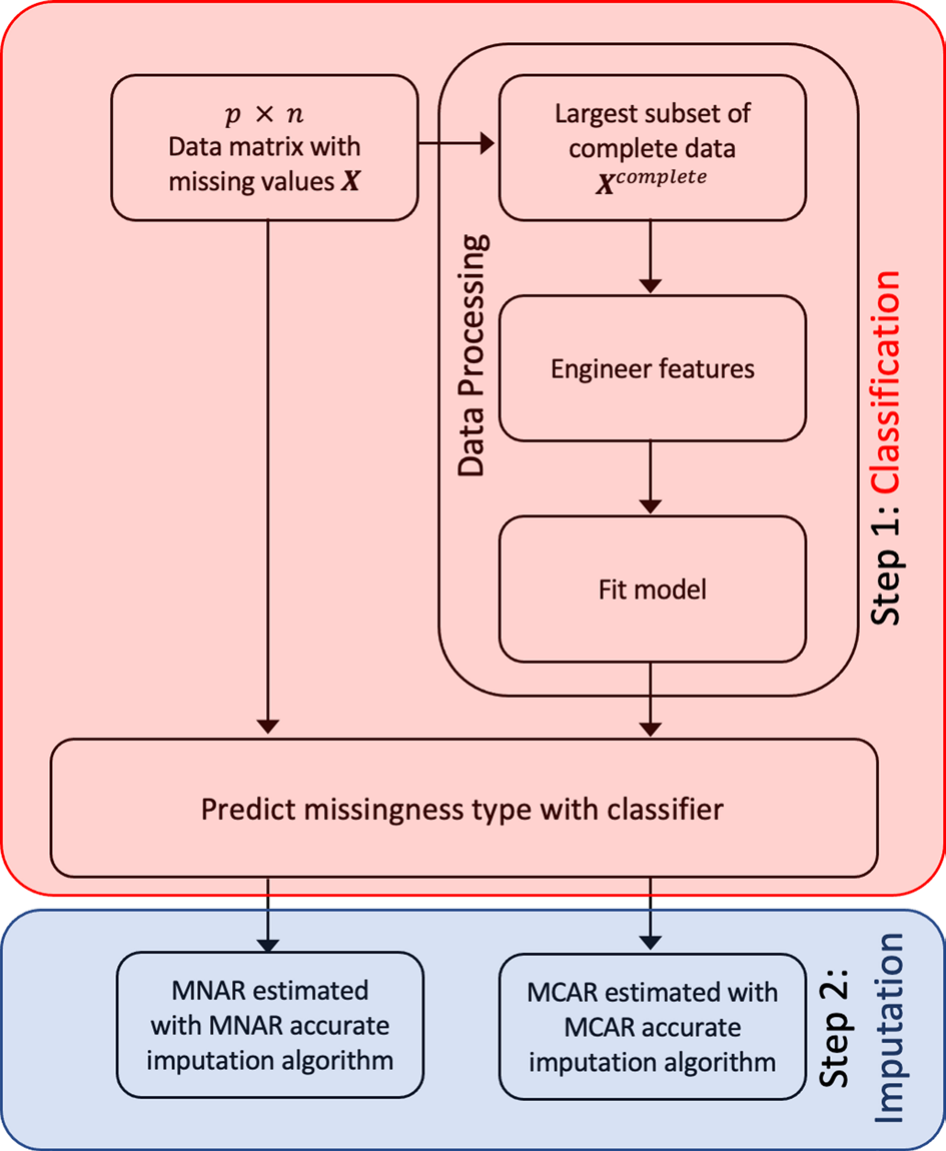
Flow-diagram of our proposed two-step, Mechanism Aware Imputation (MAI) algorithm. The red box corresponds to Step 1 of MAI for classifying the underlying missing mechanisms. The blue box corresponds to Step 2 of MAI for imputing missing values based on the type of missing mechanism as predicted in Step 1

Let ${\varvec{X}}$ be a ${\text{p x n}}$ matrix of available data containing missing values, where p is the number of metabolites (rows) and n is the number of samples (columns). We first build a random forest classifier to classify missing data as MCAR versus MNAR using a subset of complete data from our input data matrix ${\varvec{X}}$. Subsequently, we use our trained model to predict the missing mechanism in the full data matrix ${\varvec{X}}$ followed by imputing missing values using imputation algorithms specific to the predicted missing mechanisms. We depict our proposed approach in a flow diagram in Fig. 1 below.

### Building a random forest classifier

#### Fit-transform approach

The fit-transform approach is different from a traditional machine learning approach in that the new data that we wish to make predictions on is the starting data set. Moreover, the fit-transform approach draws complete data from the starting data set and transforms this complete subset into a structured data set to which we can apply traditional machine learning algorithms.

#### Generating training data

To build our classifier, we first need to extract a complete data subset from input data ${\varvec{X}}$ to generate a training data set. We elected to use a custom extraction algorithm, instead of extracting the largest block of complete data from ${\varvec{X}},$ for two reasons: (1) our extraction algorithm allows us to retain more data observations and (2) our algorithm allows us to retain all the metabolites that are present in ${\varvec{X}}$. We present a visualization of our complete subset data extraction procedure in Additional file 1: Figure S1. First, we randomly shuffle the data within each row to ensure selecting a representative range of metabolite abundances for all metabolites in ${\varvec{X}}$. Second, we move the missing values of each row to the very right end of the matrix. Lastly, we find the column index such that there are no missing values to the left of that column and extract ${\varvec{X}}^{Complete}$. The complete data subset ${\varvec{X}}^{Complete}$ will always contain all $p$ metabolites, however, the number of subjects $n^{Complete}$, where $n^{Complete} \le n$, varies depending on the missing pattern in ${\varvec{X}}$.

**Fig. 2:**
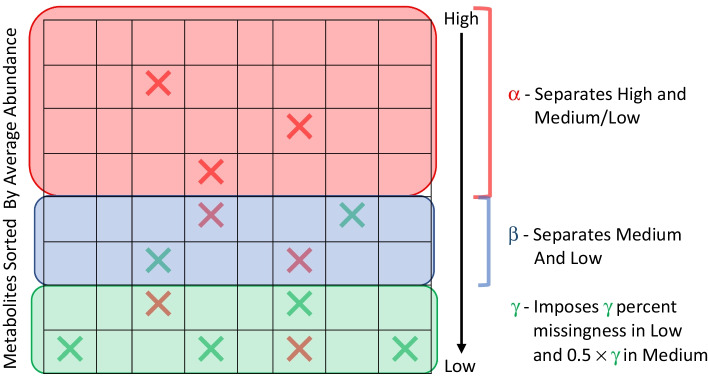
Generation of missing values within the entire data based on mixed-missingness algorithm (adapted from Styczynski et al. [9]). Rows denote metabolites and columns denote samples. The metabolites are sorted down the rows by their average abundances. The parameters α and β separate high (red), medium (blue), and low average abundance metabolites (green), while the parameter γ is used to impose missingness in the medium and low abundance metabolites. Missing values are denoted by X for MCAR (red) and MNAR (green) values respectively

After extracting our complete data subset $X^{Complete}$, we must estimate the missingness pattern within our dataset to impose missingness and generate training data for our classifier. In order to model realistic missingness patterns, we use the mixed-missingness (MM) algorithm developed by Styczynski et al. [9] in estimating and imposing missingness. The MM algorithm generates missing data according to a specified overall percent of missing values and three threshold parameters: α, β, and γ (Fig. 2). These three parameters define the distribution of MAR/MCAR and MNAR values across the dataset. The α parameter is a percentage that separates the “high” average metabolite abundance group from the “medium” and “low” average metabolite abundance groups, such that the high metabolite abundance group will not have any MNAR values. The β parameter is a percentage that separates the medium and low average abundance metabolites. The γ parameter is a percentage that generates MNAR values in the low average abundance group, while half of the γ percentage is used to generate MNAR values in the medium average abundance group. Finally, for all three groups MCAR values are generated until the prespecified total missing percentage within the entire data set is achieved [9].

To estimate the mixed-missingness parameters of our data and impose missingness on the fully observed complete data subset ${\varvec{X}}^{Complete}$, we use grid search and Euclidean distance to estimate the three thresholds, α_EST_, β_EST_, and γ_EST_, in the mixed-missingness algorithm. The purpose of estimating these parameters is to ensure that when we impose missingness in the complete data subset ${\varvec{X}}^{Complete}$ using these estimated parameters, the pattern of missingness is similar to that in *the input data matrix* ${\varvec{X}}$. We implement the grid-search via the following steps:

1. Define the grid
  - α_EST_ ranges from 5% up to the total percent missing values in ${\varvec{X}}$, in increments of 5%.
  - β_EST_ ranges from 60% up to 80%, in increments of 5%.
  - γ_EST_ ranges from 5% up to 60% in increments of 5%.
2. For each entry in the grid in step 1, impose missingness with the MM algorithm according to specified values into ${\varvec{X}}^{Complete}$ to attain ${\varvec{X}}^{Temp}$ (same dimension as ${\varvec{X}}^{Complete}$).
3. Sort the rows of ${\varvec{X}}$ and ${\varvec{X}}^{Temp}$ from high to low average metabolite abundances.
4. Calculate the proportion of missing values in each row of ${\varvec{X}}$ and ${\varvec{X}}^{Temp}$.
5. Calculate the Euclidean distance between the two vectors generated in step 4 and store the distance measurement.
6. After iterating over all the grid-values, store the α_EST_, β_EST_, and γ_EST_ parameter values corresponding to the smallest distance, as well as the distance value itself.
7. Repeat steps 2–6, 10 times and select the α_EST_, β_EST_, and γ_EST_ parameters that correspond to the smallest distance.

One iteration of this algorithm is depicted in Additional file 1: Figure S2 in the supplement.

The α_EST_, β_EST_, and γ_EST_ parameters that correspond to the smallest distance from the grid search are then used to impose classified missingness on the complete data subset ${\varvec{X}}^{Complete}$ resulting in the $p \times n^{Complete}$ data subset ${\varvec{X}}^{Imposed}$. The missingness pattern of ${\varvec{X}}^{Imposed}$ reflects the missingness pattern of input data matrix ${\varvec{X}}$, but has missing values labeled as either MCAR or MNAR according to the MM algorithm.

Our proposed algorithm used to estimate the MM parameters (Additional file 1: Figure S2) can be thought of as similar to the approximate Bayesian computation (ABC). ABC is used to attain parameters that when used in simulating data result in a data set similar to the true observed data set [12]. Similarly, ABC generates a summary statistic that is then used to compute a distance between the simulated data and the observed data [12]. However, ABC typically uses Markov Chain Monte Carlo sampling strategy to simulate plausible distributions [12]. Our approach, however, uses the MM algorithm to simulate plausible “distributions” (missing patterns).

#### Generating features for classification

Using ${\varvec{X}}^{Imposed}$ we generate features to train our random forest classifier. We generate two types of features that describe metabolites: (1) metabolite specific features that correspond to a row of ${\varvec{X}}^{Imposed}$ and (2) entry-specific features. The following features are generated:

- The mean, median, minimum, and maximum values per metabolite.
- The ratio of missing values per metabolite.
- Quantiles per metabolite that categorize each metabolite abundance value as one of 4 levels: metabolite abundances greater than the 50th percentile were designated as “high”, those between the 25th and the 50th percentiles were designated as “medium”, those less than the 25th percentile were designated as “low”, and metabolites that have been substituted by a missing indicator “MCAR”, “MNAR”, are designated as “none”. These labels are assigned for each data point.
- The non-missing metabolite abundances from ${\varvec{X}}^{Imposed}$; this is an entry-specific feature.

**Fig. 3:**
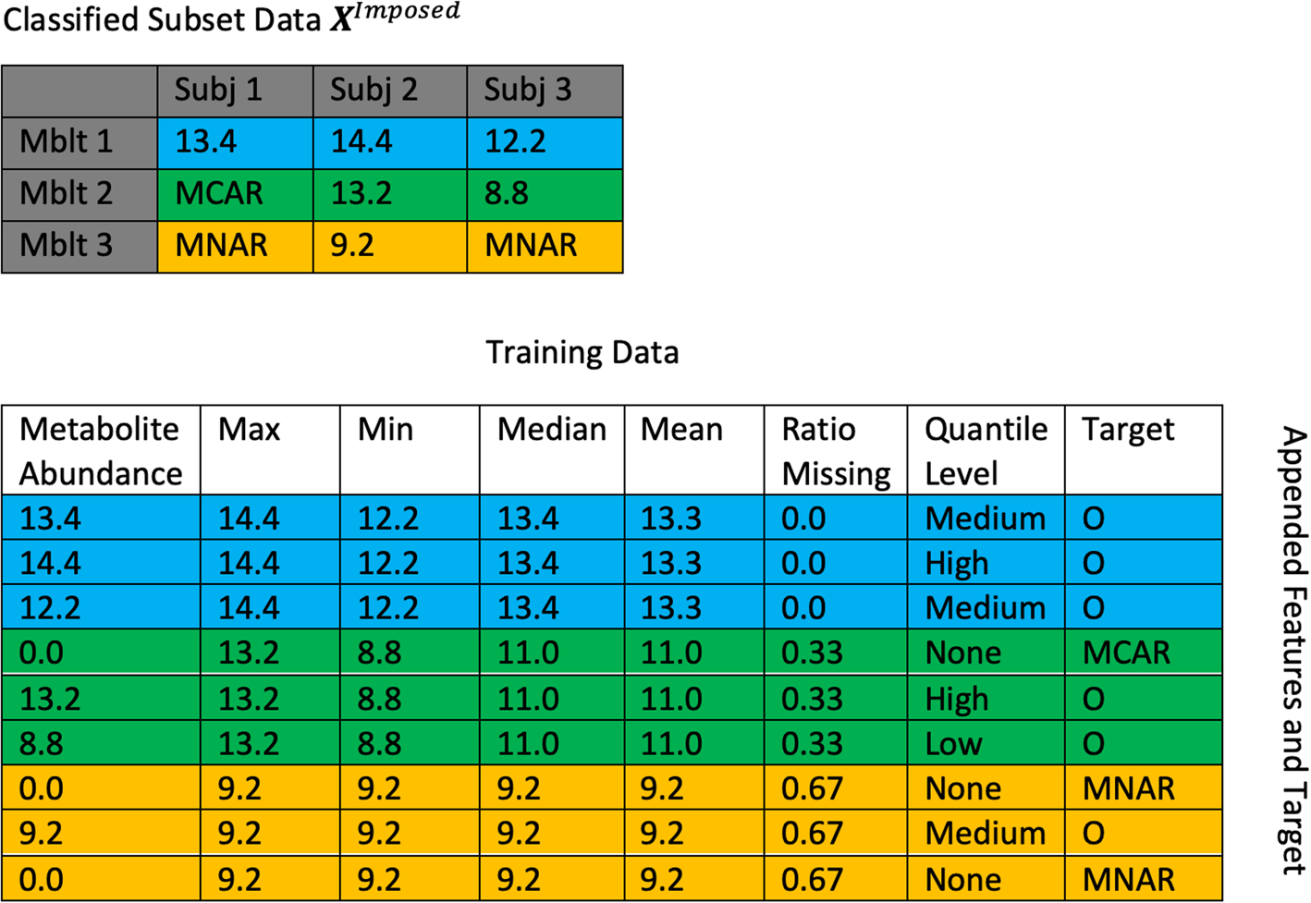
Example of training data. Classified subset data ${\varvec{X}}^{Imposed}$ (top), where rows are metabolites, columns are samples, and colors indicate different metabolites used to create the training data (bottom). Columns in the bottom table indicate the metabolite abundance, different features at the metabolite level (max, min, etc.), and quantile level for the abundance level. The class-label of interest is *Target* which is the missing data type: MCAR, MNAR, or *O* (signifies the *Othe*r nuisance class for non-missing entries). Colors indicate the original metabolites in ${\varvec{X}}^{Imposed}$ (top)

Each feature is then vectorized to collapse the $p \times n^{Complete}$ data matrix into feature-specific columns. In order to append these feature-specific columns together as a training data matrix with $p \times n^{Complete}$ rows corresponding to each entry of the data matrix, row-specific metabolite features are replicated so that they correspond to the correct metabolite row from ${\varvec{X}}^{Imposed}$. The only features that do not require replication are the quantile level categorical feature and the metabolite abundances, since those features are not metabolite-specific but entry-specific. Figure 3 depicts an example training data set that illustrates the feature generation and replication process derived from an ${\varvec{X}}^{Imposed}$ matrix.

#### Generate target column

After generating, vectorizing, and appending our feature columns, we have the final step of creating a target column to complete our training data. We define our *Target* column by duplicating the *Metabolite Abundance* column and replacing all non-missing metabolite abundances with *O*, signifying the *other* nuisance class for which data are not missing. The classes for the random forest classifier to learn are then MCAR, MNAR, and O (Other). For rows with a target of MCAR or MNAR, the metabolite abundance feature is defined as zero (Fig. 3; bottom table, first column)*.*

### Training and prediction

We fit our model by conducting two-fold cross validation while training a random forest classifier. The classifier is 300-tree deep, and the number of variables used to split at each tree is selected automatically based on the cross-validation results. We use the R programming language [13] and the caret package [14].

#### Predict missingness types in sample user input data

Using the random forest classifier trained on the features generated from ${\varvec{X}}^{Imposed}$, we classify the missingness types of our input data ${\varvec{X}}$ yielding ${\varvec{X}}^{Classified}$.

#### Impute missing values in sample user input data

**Fig. 4:**
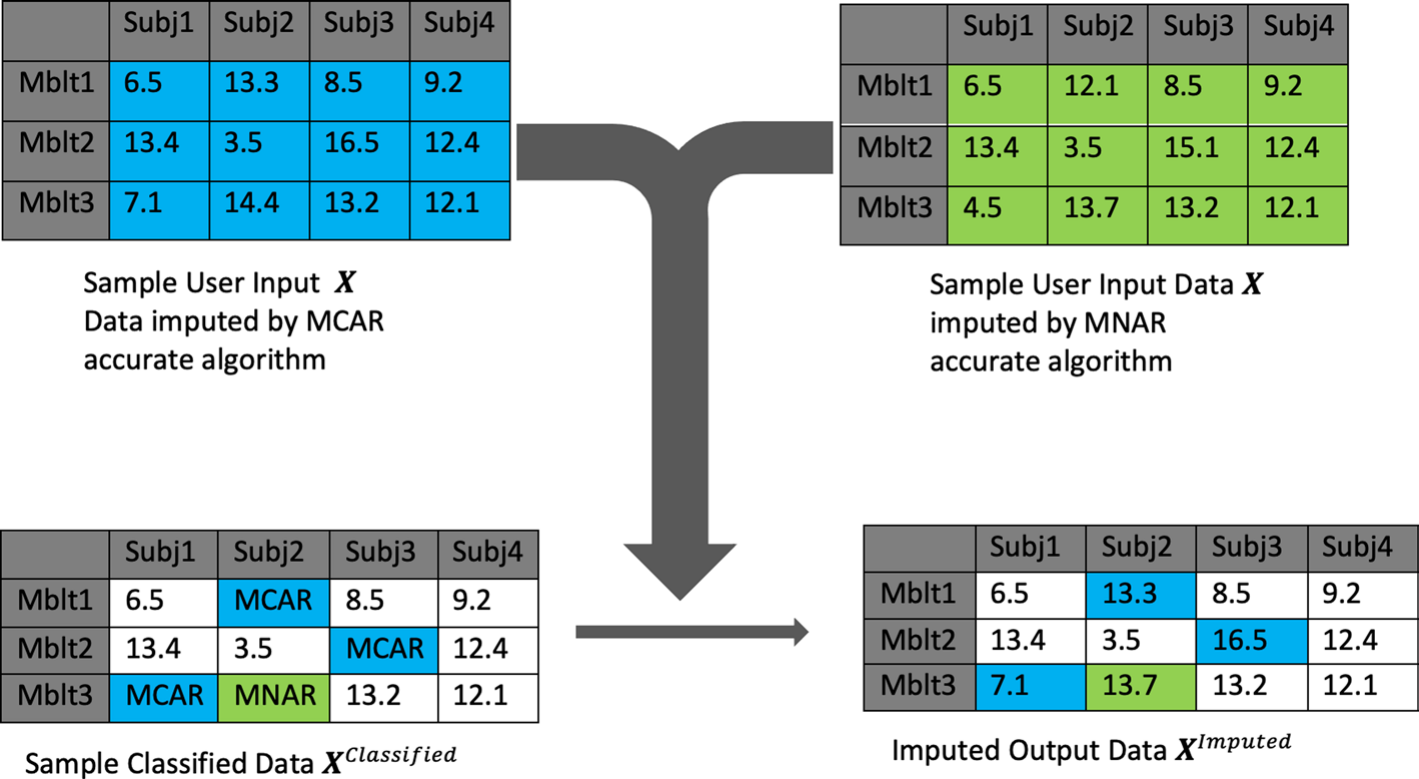
Illustration of achieving the imputed results using MAI. We first impute the input data ${\varvec{X}}$ twice (top left, top right), once with each missing mechanism-specific imputation algorithm. Subsequently, we extract the imputed values based on the predicted missing mechanism (bottom left) to construct ${\varvec{X}}^{Imputed}$ (bottom right)

Using the predicted missingness types (MCAR or MNAR), we impute the missing values using a combination of two imputation algorithms; one algorithm imputes the MCAR classified values, and the other algorithm imputes the MNAR classified values. Figure 4 depicts an example of the imputed output data*.*

### Validation

To test our method, we use two real-world untargeted metabolomics data set from the COPDGene cohort [15]. Chronic Obstructive Pulmonary Disease (COPD) is a fatal lung disease with a large global prevalence of 251 million cases in 2015 which alone was the cause of 3.17 million deaths [16]. In COPDGene Data Set 1, the fresh frozen plasma from patients in Phase II of COPDGene was profiled using the Metabolon Global Metabolomics platform via GC–MS and LC–MS metabolite quantification platforms [2]. Data were log transformed. After omitting metabolites with greater than 80% missing values across patients, the final data set contains 1243 compounds (hereinafter referred to as metabolites) and 1120 unique subjects. For evaluation, we use a complete subset of the COPDGene data without missing values: *p* = 300 metabolites and n = 100 subjects. This complete subset is our original oracle data. As a second application, we consider another plasma metabolomics data set from COPDGene. In COPDGene Data Set 2, the fresh frozen plasma from patients in Phase I of COPDGene was profiled using untargeted LC–MS (C18+) and (HILIC+) metabolite quantification platforms [17]. After omitting metabolites with greater than 80% missing values across patients, the final data set contains 662 compounds (hereinafter referred to as metabolites) and 131 unique subjects. Both data sets are available at the NIH Common Fund's National Metabolomics Data Repository (NMDR) website, Metabolomics Workbench (https://www.metabolomicsworkbench.org), with Project IDs PR000907 and PR000438 respectively. For the purpose of this work, we used versions of both data sets before any imputation was performed.

Additional file 1: Figure S3a, shows an example oracle data set where we know the original values, and S3b shows the imposed missingness derived from the MM algorithm. We use the original oracle data set to measure the accuracy of missing value imputations, and we use the imposed oracle data set to measure the accuracy of the random forest classifier.

#### Validation of random forest classification

Using the imposed oracle data and the sample classified data we validate our random forest classifier using the following accuracy metric: proportion of true missingness-type predictions divided by the total predictions made. To avoid inflating accuracy, we omit the O (other) class when reporting accuracy, since we will always get 100% accuracy for this class.

#### Performance of imputation

We implement different imputation algorithms that were recommended by Ni et al. [4] in order to compare the performance across varying missingness scenarios. Specifically, we consider the following MNAR and MCAR imputation algorithms and perform 9 combinations (3 MNAR × 3 MCAR) within the MAIs:

1. MNAR Imputation Algorithms:
  - Single Imputation
  - nsKNN
  - QRILC
2. MCAR Imputation Algorithms
  - BPCA
  - Random Forest
  - Iterative nsKNN

##### Single imputation

In a similar manner to a single imputation approach for linear regression with a randomly right-censored covariate [8], we developed a method for imputation of metabolites where left-censoring is present. Our approach assumes a multivariate normal distribution model for metabolites and estimates the expected value of a sample in presence of left censoring. Additional details of the algorithm are available in the supplement (Additional file 1: Section S3).

##### NS-KNN

This method is similar to classical KNN [18], however, neighbors with shared missing samples are assumed to be MNAR and estimated as the minimum of the sample being imputed [9]. We implemented this algorithm in the R statistical language. We select k neighbors to be the square root of the number of samples, rounded down.

##### QRILC

This method uses a quantile regression approach for the imputation of left-censored missing values [4]. We implemented this algorithm in the R statistical language using the package imputeLCMD. The parameter *tune.sigma* is used at the default value of 1, which corresponds to the case where the complete data distribution is Gaussian.

##### BPCA

BPCA uses expectation-maximization as well as a Bayesian estimation method to determine the likelihood of an estimated value [19]. We implement this algorithm in the R statistical language using the package pcaMethods [19]. The arguments, number of principal components and the number of iterations, are set at the default values of 2 and 100, respectively.

##### Random forest

Missing values are imputed by iterative fits of random forest regression model. We implemented this algorithm in the R statistical language using the package missForest [10]. We use the default parameters of 10 maximum iterations and 100-tree deep forests [10].

##### Iterative NS-KNN

We develop an iterative version of the standard NS-KNN [9]. This method uses ns-KNN for the first iteration. Subsequent iterations employ the standard KNN algorithm. At the tth iteration, for the mth metabolite-vector to be imputed (randomly chosen), we used the (m − 1) already imputed metabolite-vectors at the current tth iteration and the (p − m) imputed metabolite-vectors from the (t − 1)th iteration. We center and scale each metabolite before imputing; we also adjust the imputations considering the weighted average (using the inverse of the distances) of the k neighbors (metabolites). After imputation, we re-center and re-scale the data back to their original centers and scales. We use four iterations in total, and k is selected in the same way as in nsKNN.

To test the accuracy of each algorithm in different missingness scenarios, we selected four different γ scenarios, and fixed α and β to 30% and 70%, respectively. Recall that the α is the parameter that defines the boundary between the “high” versus “medium” and “low” average metabolite abundance groups, and the β parameter defines the boundary between the “medium” and the “low” average metabolite abundance groups. Note that due to the settings of the simulation method, once α is set there are no MNAR values introduced in the high abundant metabolites, we can change the definition of a high abundant metabolite by changing α which allows metabolites with larger mean abundances to be missing as MNAR. We introduced missingness patterns using the MM algorithm on the original oracle data with varying ratios of MCAR to MNAR values defined by our selection of γ values. These parameter values and their effects are displayed in Table 1.

**Table 1:** Effect of γ parameter on missingness patterns

γ (%)	Effect	Percentage in imposed oracle data
12	More MCAR | less MNAR	90% MCAR | 10% MNAR
23	More MCAR | less MNAR	80% MCAR | 20% MNAR
47	Moderate MCAR | moderate MNAR	60% MCAR | 40% MNAR
59	Less MCAR | more MNAR	49.5% MCAR | 50.5% MNAR

Columns indicate the effect on the ratio of MCAR to MNAR and the percentage of different types of missing data (MCAR versus MNAR) based on varying γ

We repeat the above procedure 50 times and report the means and standard errors of the normalized root mean square error (NRMSE) based on the true and the imputed values.

$$NRMSE = \frac{RMSE}{{\sigma_{Observed} }}$$

## Results

In order to evaluate our proposed approach, for each missingness scenario, we apply the two-step MAI, and separately, each individual imputation algorithm approach across 50 repetitions and report the means and standard errors of the NRMSEs based on the true and the imputed values.

### Step one of MAI achieves good classification accuracy of missing value mechanism types

The mean and median accuracies of the random forest classification model for the four simulated missingness patterns across 450 scenarios (50 repetitions × nine combinations of imputation algorithms) are depicted in Table 2. We also provide these accuracies (based on 15 repetitions) for larger total percent missing (45% and 60%) in Additional file 1: Table S7.

**Table 2:** Accuracies (across 450 simulation repetitions) of the random forest classifier in step one of MAI

	Total % missing	49.5% imposed MCAR	60% imposed MCAR	80% imposed MCAR	90% imposed MCAR
Mean accuracy [95% CI]	10%	98% [97%, 98%]	96% [96%, 97%]	93% [92%, 94%]	92% [91%, 93%]
	30%	92% [91%, 93%]	88% [87%, 89%]	82% [80%, 84%]	90% [88%, 91%]
Median accuracy [95% CI]	10%	98% [97%, 98%]	97% [96%, 97%]	93% [92%, 94%]	92% [91%, 93%]
	30%	92% [91%, 93%]	88% [87%, 89%]	82% [80%, 84%]	90% [88%, 92%]

Mean (two top rows) and median accuracies (two bottom rows) are reported for different levels of total percent missing and percent imposed MCAR missingness on the COPDGene Data Set 1; *p* = 300 and n = 100

Table 2 demonstrates that step one of two-step MAI approach is stable, in the sense that across 450 iterations the means and medians remain similar across all patterns of imposed missingness. Also, note that the step one performs comparatively better when the total percent missing is 10% (> 92% accuracy), with reasonably good performance (82–92%) for 30% the total percent missing as well. Even with more extreme value of missingness (45% and 60%, Additional file 1: Table S7), the accuracy still remains above 80% for most cases.

### MAI is robust to varying sample size and dimensions:

We evaluated effect of sample size (n) on the accuracy of the random forest classifier and tested for varying n with 30% total imposed missingness with fixed γ = 23%, α = 30%, and β = 70% (Table 3).

**Table 3:** Evaluation of the random forest classifier performance in step one of MAI for varying sample size and number of metabolites

Metabolite number and sample size combination	Mean accuracy (%)	Accuracy 95% CI	Mean NRMSE	NRMSE 95% CI
*p* = 50 n = 50	82.0	[80.1%, 83.5%]	0.260	[0.245, 0.278]
*p* = 50 n = 100	81.8	[80.3%, 83.2%]	0.264	[0.256, 0.278]
*p* = 100 n = 50	81.3	[80.2%, 82.4%]	0.282	[0.267, 0.282]
*p* = 200 n = 400	82.1	[80.0%, 83.3%]	0.259	[0.256, 0.263]
*p* = 400 n = 200	81.7	[80.0%, 82.7%]	0.272	[0.271, 0.272]
*p* = 50 n = 400	81.7	[80.2%, 82.9%]	0.270	[0.260, 0.270]
*p* = 400 n = 50	82.0	[80.1%, 83.3%]	0.273	[0.264, 0.288]
*p* = 400 n = 20	82.1	[80.1%, 82.9%]	0.239	[0.234, 0.245]

Accuracy metrics with associated 95% confidence intervals (Cis) are reported for different combinations of sample size (n) and number of metabolites (p) from the COPDGene Data Set 1

Table 3 demonstrates that the first of our two-step MAI approach is robust to different ratios of n to p. In addition, for a data set of 300 × 100 the range of X^Complete^ is expected to be in the range of [300 × 45, 300 × 51]. However, for a data set of 400 × 20 the range of X^Complete^ can be expected to be in the range of [400 × 6, 400 × 9].

### Step two of MAI achieves less biased missing value estimates:

Our proposed MAI approach, across all patterns of missingness tested, achieves the smallest average NRMSE when compared to each specific imputation approach on its own (i.e. “MCAR only” or “MNAR only”; Table 4). We focus on the results of 10% total missing here and the rest of the results are summarized in Additional file 1: Table S1 (30% total missing) and Additional file 1: Figure S4 (30% total missing simulations that achieved the most accurate imputation per missingness scenario). We also tested MAI on a COPDGene Data Set 2 and found similar results (Additional file 1: Figure S5), in addition to cases with larger total percent missing (Additional file 1: Figures S6 and S7). When γ is set to 59%, 47%, and 23% (Table 1), the combinations of algorithms that worked best for these missingness patterns are random forest imputation for MCAR values and single imputation for MNAR values. Whereas the combination of Multi-nsKNN imputation for MCAR values and nsKNN imputation for MNAR values achieved the most accurate imputations for when gamma was set to 12% resulting in 90% MCAR and 10% MNAR missingness.

**Table 4:**
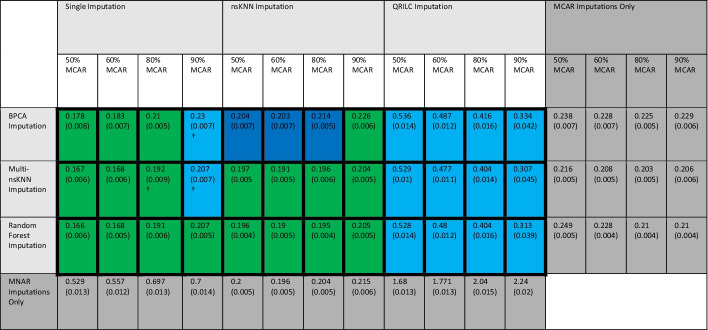
Performance of imputation methods with 10% total missing

Each cell is the mean (standard error) NRMSE across 50 simulation repetitions using the COPDGene Data Set 1 (size *p* = 300 and n = 100). Values in thicker border cells are from MAI, while the “MCAR Imputations Only” and “MNAR Imputations Only” are using one imputation method only (grey boxes). Colors indicate whether the MAI combination method is better than using only the MCAR method (dark blue), MNAR method (light blue) or both (green)

Better than both single algorithm imputations

Better than MNAR only algorithm imputation

Better than MCAR only algorithm imputation

^†^Indicates difference in means not significant at the α = 0.05 level

**Fig. 5:**
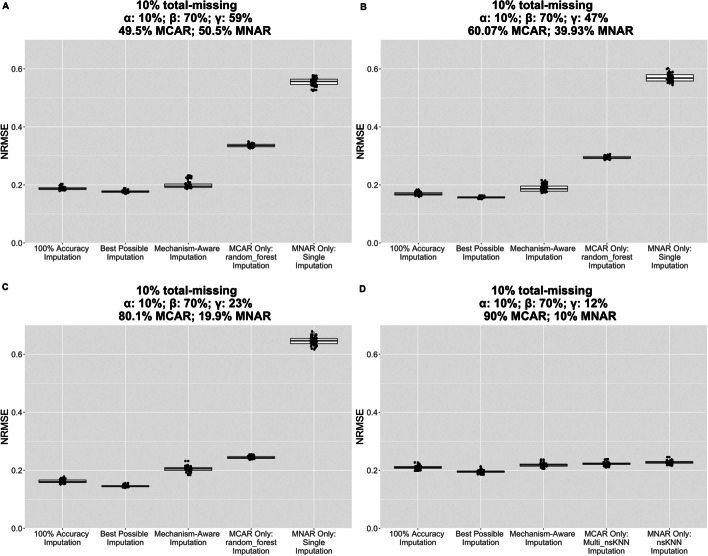
Distribution of NRMSE values for different imputation approaches for 10% total missing. **A**–**D** correspond to changing γ using the COPDGene Data Set 1 *p* = 300 n = 100. Each figure contains box plots of NRMSE (across 50 simulation repetitions) for the following approaches: *100% Accuracy Imputation*, *Best Possible Imputation*, *Mechanism-Aware Imputation*, *MCAR Only algorithm Imputation*, and *MNAR Only algorithm Imputation*. *100% Accuracy Imputation* refers to the NRMSE that would have resulted if the random forest classifier achieved 100% accuracy in step one of MAI, and the *Best Possible Imputation* refers to the NRMSE that would have resulted if the best imputed values from both the *MCAR Only algorithm Imputation* and the *MNAR Only algorithm Imputation* are selected

Figure 5 illustrates the accuracy corresponding to the best algorithm combinations for each missingness scenario when applied to the case of 10% total missing. Each panel also includes the results corresponding to two benchmark scenarios: (1) *100% Accuracy Imputation* (NRMSE if the random forest classifier identified all missingness types correctly) and (2) *Best Possible Imputation* (NRMSE if the most accurate imputation from either parent algorithm was used for each missing value).

Confidence intervals for the pairwise differences of mean NRMSEs for the three imputation methods did not include zero for the 10% total missing case (Additional file 1: Table S2), while relatively more of them did for the 30% total missing case (Additional file 1: Table S3).

### Classification accuracy in step one of MAI is dependent on alpha parameter percentage

The accuracy of the random forest classifier corresponding to the 30% total missingness scenario suggests a performance penalty due to a class imbalance (Table 2). However, even after adjusting this imbalance with synthetic minority oversampling technique (SMOTE) [20], no performance gain is achieved. Thus, we conduct a sensitivity analysis on varying the α and β parameters of the MM algorithm. We find that the performance accuracy drops as MCAR values increase due to the large α parameter value. As α approaches the total percent missing in the data set the classification accuracy drops (Additional file 1: Table S4). Next, we fix the ratio of MCAR to MNAR values in the data and then titrated the α percentages from 5 to 25% by increments of 5%. For the same MCAR to MNAR ratio, the random forest classifier has worse accuracy as α approaches the total percent missing (Additional file 1: Table S5). However, even though accuracy drops, it is always greater than 85% and the NRMSE is still smaller than using the single algorithm approach (Additional file 1: Table S1). The results of the β parameter sensitivity analysis suggest that the two-step MAI approach is robust to changes in the β parameter (Additional file 1: Table S6).

## Discussion

Our simulation results show that MAI works best when MCAR values are imputed using either random forest imputation or Multi-nsKNN and MNAR values using either the single imputation algorithm or nsKNN. Overall, we recommend using the combination of random forest imputation for MCAR values and single imputation for MNAR values for most missingness scenarios. Our bootstrapped 95% confidence intervals for the differences between the mean NRMSEs corresponding to our proposed two-step MAI approach and single algorithm approaches do not include zero (Additional file 1: Tables S2 and S3). Unsurprisingly, for all the 95% confidence intervals that include zero, the ratio of MCAR to MNAR is very large (e.g., 80% and 90% MCAR). This is because when the random forest classifier correctly identifies the missing mechanisms in step one of MAI, for the cases where MCAR is large, most of the missing entries will be imputed with the MCAR imputation algorithm. Thus, the MAI imputations for a perfect classifier will be 80% or 90% similar to those from the MCAR only imputation approach.

We point to some limitations of our proposed approach. First, the imputation algorithms used in MAI must perform commendably, otherwise the results of the two-step method will be worse than the one-step one imputation only approach. This can be seen in the imputation results (Table 2) where we use the QRILC imputation algorithm to impute MNAR values. The QRILC algorithm performs very poorly in imputing the missing values, and the effects are noticed in worse NRMSE values relative to our proposal. Second, we currently select the estimated α_EST_, β_EST_, and γ_EST_ for the MM algorithm using the smallest Euclidean distance returned from our algorithm (Additional file 1: Figure S2). However, Euclidean distances comparable to the smallest may offer other good estimates of the parameters. A potential alternative would be to implement an ensemble approach where the parameter estimates from the top *d* Euclidean distances would be used to impose missingness in the data. Third, we are limited in simulating patterns of missingness where the ratio of MNAR to MCAR is large due to the MM algorithm’s upper bound of the α parameter being the total percent missing in the entire data set [9]. Attempting to impose such a missingness pattern results in metabolites in the low average abundance group, and possibly also in some of those from the medium average abundance group, being 100% missing. Typically, metabolites that are missing greater than 80% observations are omitted from imputation due to not having sufficient information for accurate imputation. Note that this limitation is exclusive to simulation studies. In real world settings, one will simply omit the metabolites missing more than 80% observations and then utilize the MAI approach. Fourth, the mixed missingness algorithm only imposes MNAR values in the range of medium to low average metabolite abundance, assuming that MNAR values arise due to the limit of detection imposed by the measuring instrument. Finally, as with all non-naïve imputation algorithms, the effectiveness of the final imputation is dependent on proportion of non-missing data. MAI may not be as effective for a dataset with small sample size (n) or in which there is an extreme number of missing values.

Our simulations, especially those with 30% total missing case, indicate that as the random forests machine learning model learns to classify MCAR and MNAR values more accurately, NRMSE associated with imputations decrease. Therefore, in step one of MAI, future directions involve an “optimal” choice of machine learning-based classifier or the use of advanced deep learning techniques. Although we have tested our methodology exclusively on metabolomics data, our proposed approach can also be applied to other -omics (e.g., genomics, transcriptomics, proteomics) or non-omics data sets (e.g., from environmental studies where instrument-related resolution constraints are often encountered on the measurements).

The computational time of MAI is dependent on the size of the data set, the algorithms used in imputing the MCAR and MNAR classified missing values, and the total number of missing values present. For COPDGene Data Set 1 with 30% total missing it takes, on average, 2.2 min to impute MCAR values using random forest imputation and MNAR values using single imputation (1.5 min on average for 10% total missing). In contrast, using Multi-nsKNN imputation for MCAR values and nsKNN imputation for MNAR values, it takes, on average, 1.7 min and 46.1 s for 30% total missing and 10% total missing, respectively. Computational time is measured on a MacBook Pro with MacOS BigSur, 2.9 GHz 6-Core Intel Core i9 processor, and 32 GB RAM.

## Conclusion

In this article, we propose MAI—a missing-mechanism aware two-step approach—which imputes missing data more accurately than standard imputation procedures. Incorporating a random forest classifier in step one, we first predict a missing mechanism for the missing values. Then in step two, we impute those missing values using the predicted missing mechanism-specific imputation algorithms. Such mechanism-aware imputations result in better estimation of the true, unobserved (missing) data in terms of higher statistical power and less biased estimates, and consequently, more reliable conclusions derived from downstream data analyses.

### Software availability

We have developed MAI into a REST API at GitLab: https://gitlab.com/Dekermanjian/mechanismaware_imputation. We have also built a Bioconductor R package available at https://www.bioconductor.org/packages/devel/bioc/html/MAI.html. Links to both the R package and the REST API can also be found at Metabolomics Workbench: https://www.metabolomicsworkbench.org/tools/externaltools.php.

## Supplementary Information

**Additional file 1**. Additional algorithms detail, and additional simulation and sensitivity analysis results.

## Data Availability

COPDGene metabolomic data are available at the NIH Common Fund’s National Metabolomics Data Repository (NMDR) website, the Metabolomics Workbench (https://www.metabolomicsworkbench.org), study IDs ST001443 and ST000601. Data are available at the NIH Common Fund's NMDR (supported by NIH grant, U01-DK097430) website, the Metabolomics Workbench, https://www.metabolomicsworkbench.org, where they have been assigned Project IDs PR000907 and PR000438. These data can also be accessed directly via their respective project DOIs: 10.21228/M8FQ2X and 10.21228/M8FC7C.

## Abbreviations

MS: Mass spectrometry
NMR: Nuclear magnetic resonance
GC–MS: Gas chromatography coupled with mass spectrometry
LC–MS: Liquid chromatography coupled with mass spectrometry
MAR: Missing At Random
MCAR: Missing Completely At Random
MNAR: Missing Not At Random
KNN: K-nearest neighbors
BPCA: Bayesian principle component analysis
nsKNN: No-skip KNN
QRILC: Quantile regression imputation of left-censored data
MAI: Mechanism-aware imputation
MM: Mixed missingness
ABC: Approximate Bayesian computation
COPD: Chronic Obstructive Pulmonary Disease
NMDR: National Metabolomics Data Repository
NRMSE: Normalized root mean square error
SMOTE: Synthetic minority oversampling technique
