# Supplementary material for: Mechanism-aware imputation: a two-step approach in handling missing values in metabolomics
Source: BMC Bioinformatics. 2022 May 16;23:179. doi: 10.1186/s12859-022-04659-1 (PMC9109373; doi:10.1186/s12859-022-04659-1)
Supplement: Supplementary file 1 — Additional file 1. Additional algorithms detail, and additional simulation and sensitivity analysis results. [file 12859_2022_4659_MOESM1_ESM.docx]

**SUPPLEMENT**

# Mechanism-Aware Imputation: A two-step approach in handling missing values in metabolomics

Jonathan P. Dekermanjian^#*1^, Elin Shaddox^*1^, Debmalya Nandy^1^, Debashis Ghosh^1^, Katerina Kechris^1^

^1^Department of Biostatistics and Informatics, Colorado School of Public Health,
University of Colorado Anschutz Medical Campus, Aurora, Colorado, USA

#Corresponding author; email address: [Jonathan.Dekermanjian@CUAnschutz.edu](mailto:Jonathan.Dekermanjian@CUAnschutz.edu)

*Contributed equally

**Section S1: Figures**

**Figure S1.** **Illustration of complete subset (**$\boldsymbol{X}^{\boldsymbol{Complete}}$**) extraction procedure.** First, shuffle values within each metabolite (row) in $\boldsymbol{X}$. Second, shift missing values to the right end of each row in $\boldsymbol{X}$. Finally, extract the non-missing metabolite values contained in the left portion of $\boldsymbol{X}$, yielding to $\boldsymbol{X}^{Complete}$.

**Figure S2.** **Illustration of estimation procedure of the MM algorithm parameters.** The light grey box at the top depicts simulation of missing data using a combination of pre-defined MM algorithm parameters, shown in the truncated grid. The box below compares the simulated missing data in $\boldsymbol{X}^{Temp}$ to those in $\boldsymbol{X}$ using the Euclidean distance between the proportion of missing values within each metabolite (row).

A) Original Oracle Data

Subj1 Subj2 Subj3 Subj4

| 6.5 | 13.2 | 8.5 | 9.2 |
| --- | --- | --- | --- |
| 13.4 | 3.5 | 16.4 | 12.4 |
| 4.3 | 14.2 | 13.2 | 12.1 |

B) Imposed Oracle Data

Subj1. Subj2. Subj3. Subj4.

| 6.5 | MCAR | 8.5 | 9.2 |
| --- | --- | --- | --- |
| 13.4 | 3.5 | MCAR | 12.4 |
| MNAR | MCAR | 13.2 | 12.1 |

**Figure S3.** Example of A) original oracle data set (without missing values) and B) imposed oracle data (with mechanism-specific missing values).

**Figure S4.** **Distribution of NRMSE values corresponding to different imputation approaches for 30% total missing.** Figures (A)-(D) correspond to changing γ using the COPDGene Data Set 1; p=300 and n =100. Each figure contains box plots of NRMSE (across 50 simulation repetitions) for the following approaches: *100% Accuracy Imputation*, *Best Possible Imputation*, *Mechanism-Aware Imputation*, *MCAR Only algorithm Imputation*, and *MNAR Only algorithm Imputation*.
*100% Accuracy Imputation* refers to the NRMSE that would have resulted if the classifier in step one of MAI achieved 100% accuracy; *Best Possible Imputation* refers to the NRMSE that would have resulted if the best imputed values from both the *MCAR Only algorithm Imputation* and the *MNAR Only algorithm Imputation* were selected.

**Figure S5.** **Distribution of NRMSE values corresponding to different imputation approaches for 10% total missing.** Figures (A)-(D) correspond to changing γ using the COPDGene Data Set 2; p=300 and n =100. Each figure contains box plots of NRMSE (across 50 simulation repetitions) for the following approaches: *100% Accuracy Imputation*, *Best Possible Imputation*, *Mechanism-Aware Imputation*, *MCAR Only algorithm Imputation*, and *MNAR Only algorithm Imputation*.
*100% Accuracy Imputation* refers to the NRMSE that would have resulted if the classifier in step one of MAI achieved 100% accuracy; *Best Possible Imputation* refers to the NRMSE that would have resulted if the best imputed values from both the *MCAR Only algorithm Imputation* and the *MNAR Only algorithm Imputation* were selected.

**Figure S6.** **Distribution of NRMSE values corresponding to different imputation approaches for 45% total missing.** Figures (A)-(D) correspond to changing γ using the COPDGene Data Set 1; p=300 and n =100. Each figure contains box plots of NRMSE (across 50 simulation repetitions) for the following approaches: *100% Accuracy Imputation*, *Best Possible Imputation*, *Mechanism-Aware Imputation*, *MCAR Only algorithm Imputation*, and *MNAR Only algorithm Imputation*.
*100% Accuracy Imputation* refers to the NRMSE that would have resulted if the classifier in step one of MAI achieved 100% accuracy; *Best Possible Imputation* refers to the NRMSE that would have resulted if the best imputed values from both the *MCAR Only algorithm Imputation* and the *MNAR Only algorithm Imputation* were selected.

Figure S7. **Distribution of NRMSE values corresponding to different imputation approaches for 60% total missing.** Figures (A)-(D) correspond to changing γ using the COPDGene Data Set 1; p=300 and n =100. Each figure contains box plots of NRMSE (across 50 simulation repetitions) for the following approaches: *100% Accuracy Imputation*, *Best Possible Imputation*, *Mechanism-Aware Imputation*, *MCAR Only algorithm Imputation*, and *MNAR Only algorithm Imputation*. *100% Accuracy Imputation* refers to the NRMSE that would have resulted if the classifier in step one of MAI achieved 100% accuracy; *Best Possible Imputation* refers to the NRMSE that would have resulted if the best imputed values from both the *MCAR Only algorithm Imputation* and the *MNAR Only algorithm Imputation* were selected.

**Section S2: Tables**

**Table S1. Performance of imputation methods with 30% total missing.** Each cell is the mean (standard error) NRMSE across 50 simulation repetitions using the COPDGene Data Set 1; p=300 and n=100. Values in thicker bordered cells are from MAI, while the “MCAR Imputations Only” and “MNAR Imputations Only” are using only one imputation method (grey boxes). Colors indicate whether an NRMSE corresponding to MAI is better than using only the MNAR method (light blue) or both (green).

|  | Single Imputation | | | | nsKNN Imputation | | | | QRILC Imputation | | | | MCAR Imputations Only | | | |
| --- | --- | --- | --- | --- | --- | --- | --- | --- | --- | --- | --- | --- | --- | --- | --- | --- |
|  | 50% MCAR | 60% MCAR | 80% MCAR | 90% MCAR | 50% MCAR | 60% MCAR | 80% MCAR | 90% MCAR | 50% MCAR | 60% MCAR | 80% MCAR | 90% MCAR | 50% MCAR | 60% MCAR | 80% MCAR | 90% MCAR |
| BPCA Imputation | 0.223 (0.005) | 0.233 (0.006) | 0.257 (0.003) † | 0.250 (0.003) † | 0.246 (0.003) | 0.237 (0.003) | 0.246 (0.008) † | 0.250 (0.004) † | 0.814 (0.018) | 0.789 (0.017) | 0.585 (0.135) | 0.265 (0.057) † | 0.316 (0.003) | 0.29 (0.003) | 0.258 (0.003) | 0.25 (0.003) |
| Multi-nsKNN Imputation | 0.21 (0.006) | 0.218 (0.006) | 0.24 (0.005) † | 0.231 (0.004) † | 0.233 (0.003) | 0.222 (0.003) | 0.226 (0.006) † | 0.229 (0.003) † | 0.812 (0.017) | 0.785 (0.021) | 0.527 (0.146) | 0.240 (0.052) † | 0.265 (0.003) | 0.251 (0.003) | 0.236 (0.003) | 0.23 (0.004) |
| Random Forest Imputation | 0.208 (0.005) | 0.216 (0.007) | 0.238 (0.004) † | 0.230 (0.003) † | 0.232 (0.004) | 0.221 (0.003) | 0.224 (0.006) | 0.230 (0.003) † | 0.812 (0.015) | 0.783 (0.023) | 0.599 (0.14) | 0.241 (0.058) † | 0.295 (0.003) | 0.267 (0.003) | 0.238 (0.003) | 0.23 (0.003) |
| MNAR Imputations Only | 0.573 (0.01) | 0.602 (0.01) | 0.675 (0.01) | 0.725 (0.012) | 0.248 (0.003) | 0.244 (0.003) | 0.258 (0.004) | 0.273 (0.005) | 1.867 (0.01) | 1.989 (0.012) | 2.271 (0.015) | 2.450 (0.016) |  | | | |

Better than both single algorithm imputations

Better than MNAR only algorithm imputation

† Indicates difference in means not significant at the α = 0.05 level

**Table S2.** **95% bootstrapped confidence intervals for pairwise differences in mean NRMSEs across 450 simulation repetitions between all imputation approaches with 10% total missing data.** Columns represent the pairwise differences between MAI, MCAR only imputation, and MNAR only imputation. Rows represent missingness patterns grouped by the imputation algorithms used to impute MCAR and MNAR values. Computed using COPDGene Data Set 1; p=300 and n=100.

**Table S3.** **95% confidence intervals for pairwise differences in mean NRMSEs across 450 simulation repetitions between all imputation approaches with 30% total missing data.** Columns represent the pairwise differences between MAI, MCAR only imputation, and MNAR only imputation. Rows represent missingness patterns grouped by the imputation algorithms used to impute MCAR and MNAR values. Computed using COPDGene Data Set 1; p=300 and n=100.

**Table S4.** **α parameter sensitivity:** Sensitivity of random forest classification accuracy in step 1 and imputation accuracy in step 2 of MAI for total percent missing, β, and γ set to 30%, 70%, and 23%, respectively. Computed using COPDGene Data Set 1; p=300 and n=100.

| α | Total % missing | Mean Accuracy | Accuracy 95% CI | Mean NRMSE | NRMSE 95% CI |
| --- | --- | --- | --- | --- | --- |
| 5% | 30% | 96.7% | [96.2%, 97.1%] | 0.235 | [0.229, 0.241] |
| 10% | 30% | 93.2% | [92.1%, 94.3%] | 0.238 | [0.227, 0.249] |
| 15% | 30% | 90.0% | [90.0%, 91.1%] | 0.241 | [0.231, 0.251] |
| 20% | 30% | 87.0% | [85.3%, 88.8%] | 0.247 | [0.235, 0.259] |
| 25% | 30% | 84.7% | [83.2%, 86.3%] | 0.250 | [0.244, 0.256] |

**Table S5.** **α parameter sensitivity:** Sensitivity of random forest classification accuracy in step 1 and imputation accuracy in step 2 of MAI for MCAR:MNAR ratio of missing values, β, and γ set to 80:20, 70%, and 23%, respectively. Computed using COPDGene Data Set 1; p=300 and n=100

| α | Total % missing | Imposed missing MCAR:MNAR | Mean Accuracy | Accuracy 95% CI | Mean NRMSE | NRMSE 95% CI |
| --- | --- | --- | --- | --- | --- | --- |
| 5% | 5% | 80.5%:19.5% | 96.3% | [94.7%, 98.0%] | 0.178 | [0.168, 0.193] |
| 10% | 10% | 80.1%:19.9% | 93.4% | [92.4%, 94.3%] | 0.207 | [0.200, 0.215] |
| 15% | 15% | 80.2%:19.8% | 90.0% | [89.0%, 91.0%] | 0.221 | [0.212, 0.230] |
| 20% | 20% | 80.1%:19.9% | 86.8% | [83.8%, 89.8%] | 0.237 | [0.229, 0.245] |
| 25% | 25% | 80.2%:19.8% | 84.7% | [83.8%, 85.7%] | 0.244 | [0.237, 0.252] |

**Table S6.** **β parameter sensitivity:** Sensitivity of random forest classification accuracy in step 1 and imputation accuracy in step 2 of MAI for total percent missing, α, and γ set to 30%, 30%, and 23%, respectively. Computed using COPDGene Data Set 1; p=300 and n=100.

| β | Imposed missing MCAR:MNAR | Mean Accuracy | Accuracy 95% CI | Mean NRMSE | NRMSE 95% CI |
| --- | --- | --- | --- | --- | --- |
| 30% | 85%:15% | 83.43% | [80.0%, 87.1%] | 0.258 | [0.240, 0.276] |
| 50% | 82.5%:17.5% | 82.4% | [80.5%, 84.4%] | 0.258 | [0.249, 0.266] |

**Table S7.** **Accuracies (across 15 iterations) of the random forest classifier in step one of MAI.** Mean (two top rows) and median accuracies (two bottom rows) are reported for different levels of total percent missing and percent imposed MCAR missingness (columns) on the COPDGene Data Set 1; p=300 and n=100.

|  | Total missing (%) | 49.5% Imposed MCAR | 60% Imposed MCAR | 80% Imposed MCAR | 90% Imposed MCAR |
| --- | --- | --- | --- | --- | --- |
| Mean Accuracy [95% CI] | 45% | 85.9% [84.8%, 86.9%] | 80.9% [80.5%, 81.4%] | 79.3% [76.1%, 82.6%] | 90.0% [90.0%, 90.0%] |
|  | 60% | 80.2% [79.3%, 81.0%] | 75.2% [74.2%, 76.2%] | 79.5% [76.6%, 82.5%] | 90.% [90.0%, 90.0%] |
| Median Accuracy [95% CI] | 45% | 85.9% [84.8%, 86.9%] | 81.0% [80.5%, 81.4%] | 80.1% [76.9%, 83.4%] | 90.0% [90.0%, 90.0%] |
|  | 60% | 80.3% [79.5%, 81.1%] | 75.2% [74.2%, 76.2%] | 80.1% [77.2%, 83.0%] | 90% [90.0%, 90.0%] |

Table S8. Random Forest prediction model feature importances

Permutation based feature importances are ranked from most important to least in Table 4; importance scores are normalized to be between 0-100.

| Feature | Overall |
| --- | --- |
| Quantile Categories_None | 100 |
| Metabolite Abundances | 89.798 |
| Mean | 7.474 |
| Median | 6.591 |
| Proportion missing | 5.157 |
| Max | 1.571 |
| Min | 0 |

**Section S3: Additional Details on the Single Imputation Algorithm**

Our single imputation approach assumes a multivariate normal distribution for metabolites and estimates the expected value of a sample given that left censoring has occurred. Therefore, assuming $x_{j}\mathcal{\sim N}\left( \mu_{j},\sigma_{j}^{2} \right)$ where $\mu\mathbb{\in R}$ and $\sigma\mathbb{\in R>}0$, we get

$$E\left( X_{j} | X_{j}\leq c_{j} \right)=\frac{\int_{-\infty}^{c_{j}} xf_{x}\left( x \right)dx}{P\left( x\leq c_{j} \right)}$$

$$= \frac{\int_{-\infty}^{c_{j}} xf_{x}\left( x \right)\mathrm{dx}}{F\left( c_{j} \right)}$$

$$=\frac{\int_{-\infty}^{c_{j}} xf_{x}\left( x \right)dx}{1-P\left( x>c_{j} \right)}$$

$$=\frac{\int_{-\infty}^{c_{j}} xf_{x}\left( x \right)dx}{1-S\left( c_{j} \right)}$$

Expanding the numerator:

$$\int_{-\infty}^{c_{j}} xf_{x}\left( x \right)dx=\int_{-\infty}^{c_{j}} x\frac{1}{\sigma\sqrt{2\pi}}\exp\left( -\frac{\left( x-\mu\right)^{2}}{2\sigma^{2}} \right)\mathrm{dx}$$

Let $u= \frac{x-\mu}{\sqrt{2}\sigma}$ and use the error function

$$=\frac{1}{\sqrt{\pi}}\left[ \left( -\frac{-\sqrt{2}\sigma}{2}exp\left( -\left( \frac{c_{j}-\mu}{\sqrt{2}\sigma} \right)^{2} \right) \right) \right]+\frac{\mu}{2}erf\left( \frac{c_{j}-\mu}{\sqrt{2}\sigma} \right)+\frac{\mu}{2}$$

We can then solve for the expected metabolite value given that censoring occurs using the CDF or the Kaplan–Meier estimate of the survival function to use for the denominator of $E\left( X_{j} | X_{j}\leq c_{j} \right)$. In-house simulations indicated that the single imputation algorithm was more accurate using the CDF due to instability of the Kaplan-Meier estimate. So, for our single imputation application, we used the CDF in the denominator. Further, imputation was carried out by order of least missingness (i.e., the metabolite with least missingness was imputed first and the metabolite with most missingness was imputed last). Our censoring scenario was assumed to have metabolite-specific limits of detection, estimated as the minimum of each metabolite. Within a metabolite, order of sample imputation is assumed to be arbitrary.
